# A Contribution to Our Knowledge of Phytolacca Decandra, and of Grindelia Rocusta

**Published:** 1877-11

**Authors:** 


A Contribution to our Knowledge of Phytolacca Decandra, and of Grindelia Rocusta.—1. *Phytolacca*—Everybody is familiar with the appearance of the despised poke, but few are aware of its really valuable properties. No systematic study has hitherto been made of its physiological actions. The accidental administration of poke-berries, in sufficient quantity to cause serious symptoms, has furnished information of great value. I have studied these cases, to compare the symptoms produced in animals, with those which have occurred in man.

It has long been known that poke is a nauseant and emetic, and that vertigo, convulsions and coma have been observed in cases of poisoning by this substance. Referring to these symptoms, the United States Dispensatory remarks that it must possess "unsuspected powers" as a "spinal irritant." The accounts which have been published of cases of accidental poisoning, show that poke affects the functions of both brain and spinal cord.

My experiments were made on frogs and rabbits. The most characteristic reactions are produced in these animals; and as these reactions correspond to the symptoms observed in man, we may accept the results without hesitation.

Phytolacca is a most depressing and nauseating emetic, and has been proposed as a substitute for ipecacuanha; but the suggestion has never been acted on, because the action is so intensely disagreable. Both in frogs and rabbits the nauseant effect is very obvious. When the frog is nauseated by it, the under-jaw drops, and he presents the most ludicrous appearance of intense disgust. Phytolacca is a specific emetic—that is, whether taken into the stomach or thrown under the skin, it causes nausea and vomiting. The gastro-intestinal secretions are increased by phytolacca. Diffusion into the blood takes place with facility; but in what form, and what changes, if any, are induced by it in the composition of the blood, are quite unknown. It slows the cardiac movements and lowers the arterial tension. The action of the heart continues after the entire cessation of the respiratory movements. This can be easily seen as follows: Paralyze a frog with it until all external signs of life have ceased, when, on opening the chest, the heart is still seen in action. But it is a heart poison as well as a respiratory poison, although it affects the respiration more than the heart. Phytolacca affects warm-blooded animals similarly. When paralytic symptoms are produced in a rabbit, pass into the heart, through the chest-wall a long, strong needle having a rod of pine-wood attached. Every movement of the heart is then represented to the eye by the movement of the rod. It is obvious that any subsequent changes in the cardiac movements will rupture the synchronism between the rod and the metronome. I have made a number of observations on the action of the frog's heart, with the metronome, of which the following experiment is an example:

Injected a large frog, intended for the table, with ʒj of the fluid extract. The first effect was sluggishness in all the voluntary movements, with increasing paresis. When a fore extremity was doubled up under the chest, he made no attept to remove it. The paresis increased, so that he could not jump, and the hind extremities were partly extended. The lower jaw dropped, and the mouth remained widely open. When now, an extremity was sharply pinched, he manifested no sign of pain. In an hour there was complete muscular relaxation, and all external signs of life had ceased. On opening the chest, by dividing the sternum, the heart was found to be in action feebly, beating eighteen per minute. Adjusting the metronome to strike at the same rate, the action of the heart was ascertained to be slowing and its pulsations feebler.

At the end of two hours the effect of the dose had declined considerably; the frog made violent efforts to turn over, and at last succeeded. The action of the heart then rose to 30. When he had turned over and assumed the usual sitting posture, he looked quite natural notwithstanding the opening in the chest.

The experiment just narrated apparently demonstrated that phytolacca is a paralyzer of motility and sensibility. Exact observation on the sciatic nerve, the limb being isolated and separated entirely from the body, except by the nerve, showed that the sensibility of the sensory and motor nerves to their appropriate stimuli, was unaffected.

The paralysis of motion and of sensation is therefore due to an impression made by phytolacca on the spinal cord.

In a young rabbit weighing forty-six ounces, a drachm of fluid extract of phytolacca administered subcutaneously, caused at first excitement followed by stupor, weakness of the extremities, trembling of all the voluntary muscles, and especially trembling of the ears; increasing paralysis, both motor and sensory, contracted pupils, convulsions, death ensuing from failure of respiration. A number of trials on rabbits produced essentially the same results. I need not, therefore, occupy time in detailing them.

The therapeutical applications of phytolacca are, as yet, strictly empirical. A good deal of clinical experience has been accumulated—most of it to be found in the United States Dispensatory—showing that poke possesses properties to which the old and meaningless term, *alterative*, has been applied. The berries, usually steeped in whisky, have been employed with success in the treatment of *chronic rheumatism.* Whether berries or whiskey has been most influential in bringing about a cure, has not yet been determined. So many instances of good effects following its use in chronic rheumatism have now been reported, that it must be regarded as having some special power over this affection. Cases of *constitutional syphilis* have, it is said, been arrested by the use of phytolacca. (Resources of Southern Fields and Forests, p. 402.) Locally applied in the form of decoction, *parasitic skin diseases*, unhealthy *wounds*, *ulcers*, and even *cancer*, have been reported cured.

While many of these alleged cures are no doubt apocryphal, there must be some fact as a basis for pretensions so positive. As respects the supposed alterative powers of poke, they may be regarded as similar to those of guaiac. Indeed, all remedies which increase the secretions of the intestinal canal, kidneys and skin promote elimination of all foreign and excrementitious materials which seek an outlet through these channels. The good results which have followed the application of a decoction of poke to unhealthy wounds, may well be admitted as genuine, for decoctions of other astringents are used with advantage in such cases.

Thus far, it must be admitted that the empirical knowledge of the uses of phytolacca is not extensive, and doubtless far from accurate. There is, however, another application of this agent, which, if sustained by future experience, must be regarded as extremely important. I refer to its use in *mastitis*, inflammation of the mamma. Dr. Tidd, not long since, published, in *The Clinic*, a paper showing the utility of phytolacca in this disease—showing, indeed, that it has the remarkable power to prevent suppuration. Others have published similar experiences, and I am favored with a verbal communication from my colleague, Prof. Palmer, in which he expressed a strong conviction that phytolacca really does possess this remarkable property. If it shall be settled that mastitis may be arrested, and suppuration prevented, by the use of this remedy, it is plain that we have in poke a drug of very unusual power. I do not conceal from myself the great difficulty of discriminating between the *post hoc* and the *propter hoc* in such a malady as an inflamed breast proceeding to suppuration. Numerous observations must be made, and under conditions that admit of no misconception, before this question can be settled.

2. *Grindelia.*—This remedy comes to us from California. Its botanical name is grindelia robusta, and the leaves, stems and flowers are the parts employed in preparing pharmaceutical products. The most eligible preparation is the fluid extract.

Grindelia has not, as yet, been subjected to thorough chemical investigation. Partial examination has shown that it contains an alkaloid, having basic properties. The oleo-resin is probably an important constituent also.

As considerable demand has sprung up, already some sophistication has been practiced, other members of the family having been used for this purpose.

My experiments were made on frogs and rabbits, chiefly, and I supplemented these by some observations on myself to a limited extent. Besides these sources of information, I have consulted all the papers which have been written in regard to its effects on certain diseases, and have prescribed it in maladies, for the relief of which it is so highly commended by our Californian colleagues.

Grindelia is by no means actively toxic, since a half-ounce of the fluid extract was required to kill a small rabbit. The taste is bitter, rather acrid, and is decidedly persistent. In the stomach, in any considerable quantity, it gives rise to a sensation of warmth, which diffuses throughout the system. The action of the heart becomes more energetic, the tone of the vascular system rises, and increased secretion, salivary and cutaneous, takes place. The increased blood pressure is not long maintained, but descends a little; the respiratory movements, at first hastened a little, become slower and fuller.

The most important action of grindelia, unquestionably, is its influence on the cerebrum. The first impression is to increase the activity of the cerebrum, but this stage of excitation is soon succeeded by a condition of mental calm, lapsing into sleep, but not into coma. During this stage of sopor the pupils are somewhat dilated, the respirations become slow, but are deeper, and the rythm continues normal. In rabbits the condition of sopor is accompanied by a marked degree of muscular paresis, beginning in the hind extremities. The contractility of the muscles and the irritability of the nerves, both motor and sensory, remain unimpaired, so that the paralytic symptoms are due to the action of the agent on the brain or spinal cord, or both.

Notwithstanding grindelia produces distinct paralytic effects, it exalts the reflex functions of the spinal cord in a remarkable manner. When a frog lies with all the limbs extended, and deprived of all power of voluntary movements, a gentle tap on any part of the body is followed by rapid, tetanic contractions of all the voluntary muscles, beginning in the upper extremities, next extending to the spinal muscles, and reaching in a second of time the muscles of the leg. These tetanic spasms, produced by a peripheric irritation, seem to me to be of the same character as those which occur in frogs after some hours of complete muscular resolution, caused by lethal doses of atropia.

Death ensues by paralysis of the muscles of respiration, and cavities of the heart (frogs) are distended with blood. The elimination of grindelia takes place through the kidneys chiefly. The oleo-resin is more or less irritating to these organs; more frequent irritation results, and the amount of urine discharged is increased above the normal. As respects the action of grindelia on the kidneys, it is comparable to that produced by buchu, uva ursi, eucalyptus, and other agents whose activity is due to the presence of an oleo-resin. A peculiar odor is imparted to the urine by the oleo-resin of grindelia.

The therapeutical applications of grindelia might be deduced from a study of its physiological actions. The combination of an oleo-resin with a narcotic principle, is one especially advantageous in the treatment of the respiratory neuroses. The same result has been reached empirically. Our Californian colleagues have used grindelia with success in the treatment of the asthmatic paroxysm. If I may form an opinion from an experience with a few cases, I must admit that the California reports are well founded. In several cases of asthma, in cough maintained by habit, and in spasmodic cough from reflex causes, it has afforded decided relief. Like other so-called expectorants and diuretics, which contain an oleo-resin, grindelia is serviceable in chronic bronchitis, and bronchorrhœa, and in catarrhal states of the genito-urinary mucous membrane.

The effects which grindelia produces on the cerebrum, as a hypnotic and anodyne, are strongly suggestive of a sphere of usefulness in the future. How far the anodyne and hypnotic action, so conspicuous in animals, will occur in man, remains to be seen.—*Journal of Nervous and Mental Disease.*

